# Reinforcement Learning With Parsimonious Computation and a Forgetting Process

**DOI:** 10.3389/fnhum.2019.00153

**Published:** 2019-05-09

**Authors:** Asako Toyama, Kentaro Katahira, Hideki Ohira

**Affiliations:** Department of Psychology, Graduate School of Informatics, Nagoya University, Nagoya, Japan

**Keywords:** decision-making, reinforcement learning, computational model, model-based learning, action sequence, forgetting process

## Abstract

Decision-making is assumed to be supported by model-free and model-based systems: the model-free system is based purely on experience, while the model-based system uses a cognitive map of the environment and is more accurate. The recently developed multistep decision-making task and its computational model can dissociate the contributions of the two systems and have been used widely. This study used this task and model to understand our value-based learning process and tested alternative algorithms for the model-free and model-based learning systems. The task used in this study had a deterministic transition structure, and the degree of use of this structure in learning is estimated as the relative contribution of the model-based system to choices. We obtained data from 29 participants and fitted them with various computational models that differ in the model-free and model-based assumptions. The results of model comparison and parameter estimation showed that the participants update the value of action sequences and not each action. Additionally, the model fit was improved substantially by assuming that the learning mechanism includes a forgetting process, where the values of unselected options change to a certain default value over time. We also examined the relationships between the estimated parameters and psychopathology and other traits measured by self-reported questionnaires, and the results suggested that the difference in model assumptions can change the conclusion. In particular, inclusion of the forgetting process in the computational models had a strong impact on estimation of the weighting parameter of the model-free and model-based systems.

## Introduction

Computational models are tools used to understand decision-making processes. One successful model designed for this purpose was developed by Daw et al. (2011) and can dissociate the contributions of two value-based learning systems to choice behavior. One such system is the model-free system in which values are incrementally learned through direct experience. The other system is a model-based system in which values are calculated using a “model,” or cognitive map, of the environment (Tolman, 1948) to calculate its values. The relative contributions of these systems have been estimated by applying computational models or logistic regression models to data from the two-step decision task (Daw et al., 2011). The framework of the two learning systems has enabled productive discussions and has helped to construct theories in cognitive, psychopathological, and neuroscience research by revealing developmental changes in model-based weight (Decker et al., 2016), the predominance of model-free choice under certain circumstances (Otto et al., 2013), the neural basis of the model-free and model-based systems (Smittenaar et al., 2013; Daw and Dayan, 2014; Lee et al., 2014) and the relationships of the two systems with clinical symptoms such as those observed in obsessive-compulsive disorder (OCD) (Voon et al., 2015; Gillan et al., 2016).

The widely used computational model for the two-step decision task (Daw et al., 2011) provides a favorable parameter allowing prediction of stress conditions or OCD tendencies. However, to increase our understanding of cognitive processes, we still need to examine alternative algorithms of the model-free and model-based systems other than those used in the widely used computational model. For example, although the model-free and model-based values are often assumed to be calculated in parallel, some studies have suggested the existence of an interaction between the model-free and model-based systems (Dezfouli and Balleine, 2013; Gershman et al., 2014; Toyama et al., 2017). In addition, differences in model construction may substantially influence parameter estimation (Katahira, 2018) because when a computational model is applied to data, the data can be explained only by adjusting the parameters under the framework of the model. Therefore, improving the model data fit can diminish possible noise and biases in parameter estimation and minimize undesirable, misleading results. Thus, the purpose of this study is to examine alternative hypotheses regarding the model-free and model-based systems by comparing candidate computational models with different algorithms.

In this study, we used the two-step decision task developed by Kool et al. (2016), which is a variation of the task developed by Daw et al. (2011). This task has a few desirable features. First, the model-based weighting parameter, which is a key parameter in the computational model, seems to be relatively easy to interpret when estimated from the data obtained during this task. In the original two-step task, participants learn and use a stochastic action-state transition structure (the “model” of this task); therefore, this weighting parameter reflects the degree of learning of the model in addition to the degree of willingness to use it. On the other hand, the Kool two-step task has a deterministic transition structure where each action leads to specific subsequent state in a deterministic manner, which is relatively easy to learn for participants. Therefore, the model-based weighting parameter is presumed to reflect greater use of the model. In addition, the simplicity of the Kool two-step task in other respects^1^ can also suppress unexpected strategies that participants may use to make choices and can reduce the noise in the parameter estimates given by a computational model. This advantage is critical because a computational model cannot treat all possible strategies or intentions^2^. Therefore, we selected the Kool two-step task to develop a computational model with reduced noise and to consider the algorithms underlying model-free and model-based decision-making. Specifically, using this task, we examined the computational assumptions that we proposed in Toyama et al. (2017) for the Daw two-step task and an additional assumption unique to learning in tasks with a deterministic structure, such as the Kool two-step task. We will explain these assumptions in detail after we outline the procedure of the Kool two-step task.

**Figure 1:**
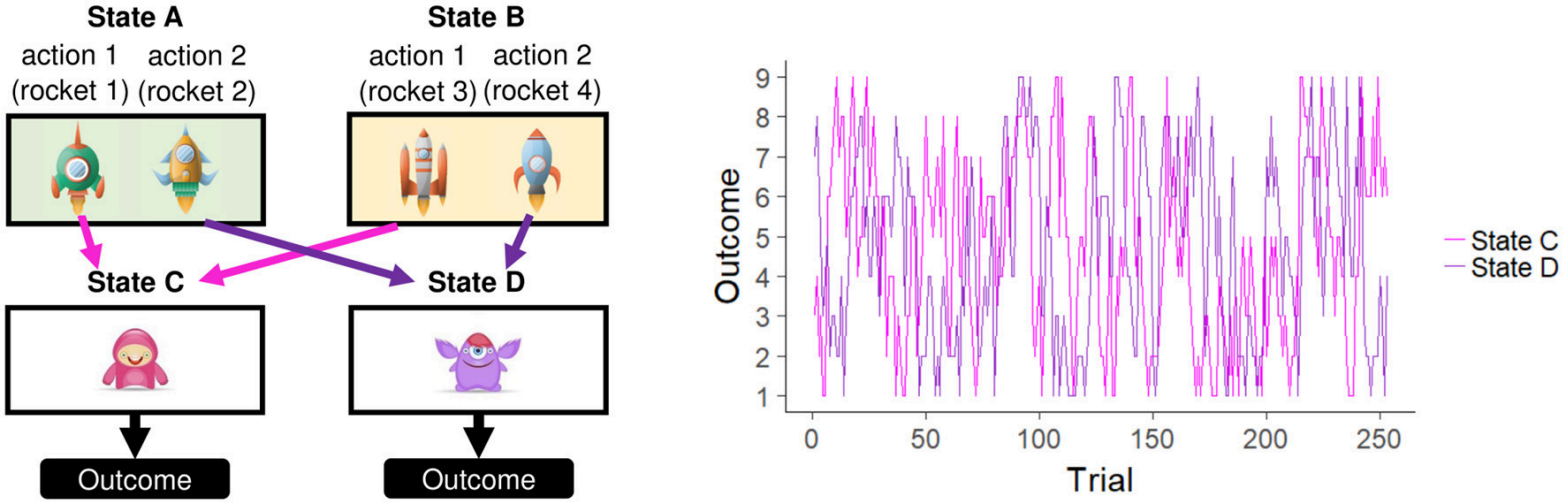
The two-step task used in the experiment **(left panel)** and its outcome design **(right panel)**. Two stages were included in each trial. The first stage started with one of the two states: state A, which included two rockets, or state B, which included two other rockets. The participants selected one of the two rockets, which deterministically led to a specific second-stage state (action 1 to State C and action 2 to State D). After the subject pressed the spacebar key in the second-stage state, the outcome reward was displayed as an integer point value ranging from 1 to 9. This point value changed slowly and independently according to Gaussian random walks, but the same values applied to all participants.

In the Kool two-step task, participants are required to choose an action (i.e., choose a rocket) in the first stage, which is followed by a second-stage state (a screen with an alien) and a reward outcome (Figure 1). The participants' goal is to maximize the total reward amount. In each trial, the first stage is shown as one of the two states, including two options (state A with rockets 1 and 2 and state B with rockets 3 and 4). A key feature of this task is that one of the rockets in each state is always followed by a specific second-stage state, while the other rocket is always followed by the other second-stage state (rockets 1 and 3 always lead to second-stage state C, and rockets 2 and 4 always leads to second-stage state D). Thus, this task encourages participants to use the task structure, or “model,” to base their choices on their past outcome experiences. For example, when a participant wins a large reward in a previous trial but the current first stage is different from the previous first stage, the participant must use the model of the transitions to consider which rocket leads to the previously experienced second-stage state.

**Figure 2:**
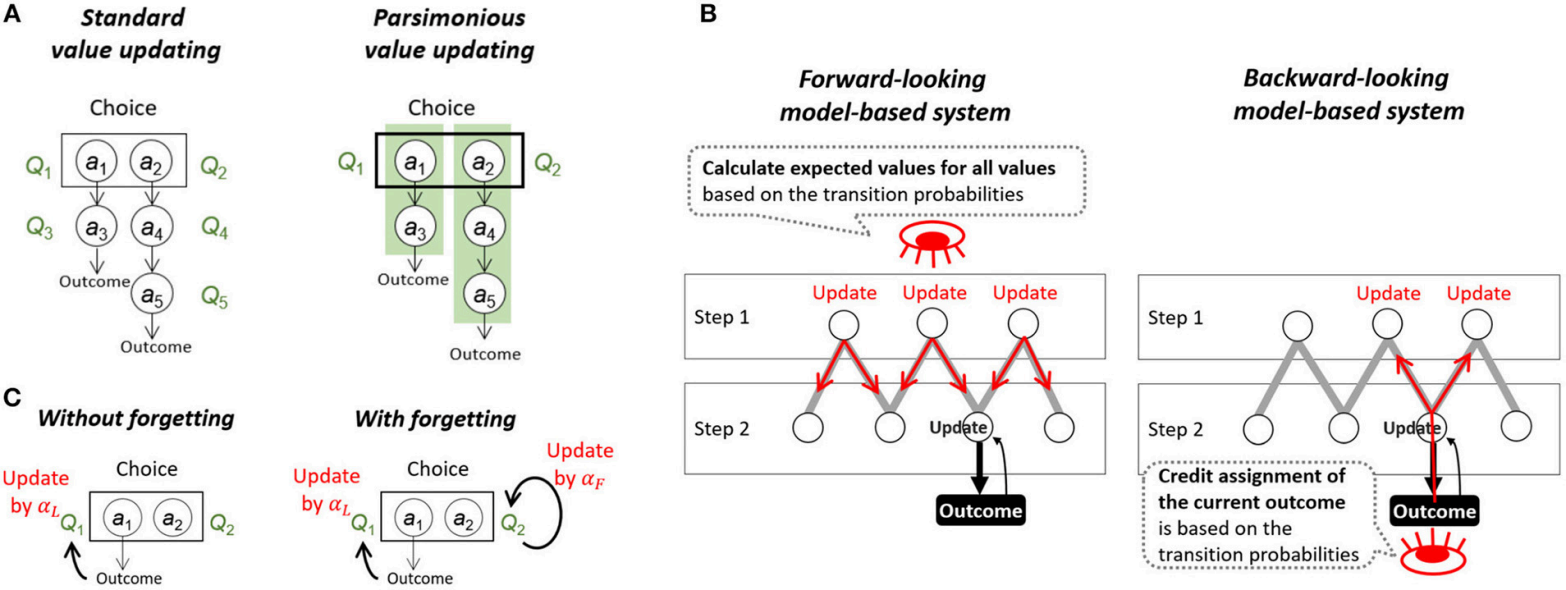
A conceptual framework for the examined assumptions. **(A)** The standard model-free algorithm is the SARSA temporal-difference (TD) learning model, where the values are computed for all state-action pairs (standard value updating). We examined another possibility in which only the action values in the choice stage are computed (parsimonious value updating). **(B)** The originally used model-based system assumes that the expected values for all state-action pairs are calculated anew each time using the transition-probability model of the task (the forward-looking model-based system). This system carries a high calculation cost but realizes fully model-based updating. As another possibility, we applied model-based updating for the credit assignment problem (the backward-looking model-based system). This system updates only the state-action pairs relating to the last state that produced the outcome based on the transition-probability model of the task, but it works efficiently with similar accuracy to the forward-looking model-based system when the transition probabilities are stable. **(C)** In the standard TD learning algorithm, the values of unselected options are assumed to remain unchanged (without forgetting). We examined another possibility in which the values of unselected options change to a certain default value over time (with forgetting).

Using this task, we examined several reinforcement learning (RL) models to express the integrated algorithm of the model-free and model-based learning systems. The assumptions that we examined in the new models are inspired by psychological considerations. First, we considered cognitive savings regarding the values to be updated during learning. Deterministic probability is a special case of stochastic probability; however, if the transition is deterministic, we do not need to discriminate successive actions. The algorithm of the model-free system is typically the SARSA (state-action-reward-state-action) temporal-difference (TD) learning model (Rummery and Niranjan, 1994), where the values are updated for all experienced state-action pairs (Figure 2A, left). However, if a choice is deterministically followed by certain state-action pairs, a parsimonious computational algorithm where only the value of the first action is computed can be assumed (Figure 2A, right). In the Kool two-step task, computing the values of first-stage actions is sufficient because the subsequent actions in the second stage are deterministic. Additionally, cognitive resources can be saved by using this type of chunk unit (Miller, 1956).

When a computational model uses the above parsimonious computational algorithm, a typical model-based system is impossible to apply because the typically used model-based system is a *forward-looking* system, calculating the expected values of each action using the Bellman equation for the estimated values of the future step (Figure 2B, left). Thus, we applied a *backward-looking* model-based system similar to the system that we applied to the data from the Daw two-step task (Toyama et al., 2017). The *backward-looking* model-based system assumes that “credit assignment of the outcome” is implemented in a model-based manner (Figure 2B, right). The impact of the outcome reward directly updates the actions that can lead to the last state that produces this outcome. The advantage of this model-based updating is that participants do not need to activate the representations of all state-action values but only the values of states and actions associated with the last state.

In addition, regarding the model-free system, we applied the concept of memory decay in the model-free part of RL. In the standard TD learning algorithm, the values of the unselected options are assumed to remain unchanged (Figure 2C, left). However, this assumption is unnatural given that memory decays over time (i.e., forgetting). A few studies have introduced a learning model with a forgetting process (Figure 2C, right) in which the unselected option values are assumed to gradually approach zero (Barraclough et al., 2004; Ito and Doya, 2009). Our previous study (Toyama et al., 2017) extended this model by adding a new parameter, “default value,” such that we could freely set the endpoint of forgetting instead of restricting it to zero. The default value represents an expected value for options in the absence of knowledge or experience related to the relationship between options and outcomes.

Overall, the examined computational models have some or all of these assumptions. These models were compared using data from the Kool two-step task. In addition, to test the effect of model construction on the parameter estimates, we compared the computational models in terms of the relationship between the estimated parameter values and subjects' scores on questionnaires regarding obsessive tendencies, impulsivity, and other psychological features.

## Materials and Methods

### Participants

Thirty-four undergraduate students at Nagoya University participated in the experiment. The data from two participants were excluded because the participants were unable to complete the training session by themselves due to their misunderstanding of the instructions, and three participants were excluded because they did not pass the exclusion criteria (see section Exclusion criteria). Thus, the data from the remaining 29 participants were analyzed (13 males, 16 females; age *M* = 18.7 years, *SD* = 0.9). All participants provided written informed consent in accordance with the Declaration of Helsinki. The protocol was approved by the ethical committee of Nagoya University.

### Apparatus

Participants were seated ~50 cm in front of a 21.5-inch iiyama ProLite monitor with a screen resolution of 1920 × 1080 pixels and a refresh rate of 60 Hz. Instructions and stimuli were presented using the computer program Inquisit 5 Lab (2016) by Millisecond Software in Seattle, Washington.

### Two-Step Task

The task procedure was almost the same as the two-step task originally proposed by Kool et al. (2016, 2017), although we slightly changed the cover story and settings. The participants' goal was to maximize their reward by their choice of rockets.

Each participant completed 253 trials, which were divided into two blocks separated by 30-s breaks. Each trial consisted of two stages. In the first stage, the participants were required to select one of two rockets (downloaded from Freepik.com) by pressing the F key for the left rocket or the J key for the right rocket within 2.5 s. This stage was characterized by one of two states: state A always included rockets 1 and 2, and state B included rockets 3 and 4. The subsequent second-stage state was based on the first-stage choice. Rockets 1 and 3 were always followed by state C in the second stage, and rockets 2 and 4 were always followed by state D in the second stage. In the second stage, each state included one unique alien (downloaded from pngtree.com). The participants were required to press the space bar within 1.5 s to obtain a reward from the alien. Each alien produces a reward feedback value ranging from 1 to 9. These feedback values for each alien changed slowly over the course of the task according to a Gaussian random walk (mean = 0, σ = 0.025) with bounds of 0.25 and 0.75 and was displayed as an integer on the screen. Auditory stimuli were played when participants made a choice (bell sound) and when they obtained a reward (money sound).

At each stage, if no response was made within the time limits, a message reading, “Too late!!” was presented, and the participants proceeded to the next trial.

### Instructions and Training Session Before the Task

Before the task, the participants were informed that the positions of the rockets and the response speed within time limits would have no relationship with subsequent feedback or the total experimental time and that the choice of rockets is only related to the transition to the second-stage states. The participants were also repeatedly told that each rocket in each first stage was connected decisively with one of the two aliens in the second stage and that the reward from each alien would change slowly and independently over time depending on these aliens' moods within the range from 1 to 9. Thus, the participants were informed that they would obtain greater rewards by focusing on the moods of each alien. The participants were also informed in advance that they could receive additional monetary rewards along with their total earned points in this task. Specifically, the participants were paid ¥1,000, with an additional monetary reward of either ¥300 (if they earned more than 1,300 points) or ¥200 (if they earned fewer than 1,300 points).

The participants also completed a training session to learn the structure of the task in advance; in this session, they were required to repeatedly choose the rockets connected with one of the two aliens in the training trials without time limits or feedback, and if they succeeded in more than 5 consecutive trials for each alien, then they were next trained with 18 trials with time limits and feedback. The stimuli used in the training session were completely different from the stimuli used in the real task.

### Task Settings

The reward probabilities were the same for all participants, but the order of the first-stage state during the task was deliberately controlled in advance, and each participant was allocated to one of four sequences (see Supplementary Text 1.1, Figure S1 and Table S1). The full series of 253 trials started with one of the two first-stage states, and the same first-stage state was repeated within 6 trials. Except for the first trial, 180 trials started with the same first-stage state as the previous trial, and the remaining 72 trials started with a different first-stage state from the previous trial. In the former class of trials, participants do not need to use the transition model, whereas in the latter class of trials, participants need to use the transition model if they wish to use the information from previous feedback. For the analysis in the results section, we conveniently refer to the former trials as *MF (model-free) trials* and the latter trials as *MB (model-based) trials*. The choices predicted by the model-free and model-based systems are similar in the MF trials but not in the MB trials.

### Exclusion Criteria

In the analyses, we excluded the data from uncompleted trials (i.e., those in which the choice was not made within 2.5 s) and the data from trials in which the response time was <120 ms, which were considered anticipated responses that did not reflect the stimulus types. Two participants who had more than 20% of their trials omitted based on these criteria were excluded. In addition, we excluded one participant who chose the same rocket in each first-stage state in more than 90% of the trials. Thus, the data of 29 participants were used for the subsequent analyses (rate of excluded trials: max 8%, mean 1%).

### Questionnaires

After the two-step task, the participants completed the Japanese versions of several questionnaires. OCD tendencies was assessed using the Obsessive-Compulsive Inventory (OCI) [Foa et al., 1998, Japanese version: Ishikawa et al., 2014], depression was assessed using the Self-Rating Depression Scale (SDS) (Zung, 1965; Japanese version: Fukuda and Kobayashi, 1973), trait anxiety was examined using the trait portion of the State-Trait Anxiety Inventory (STAI) (Spielberger et al., 1970; Japanese version: Shimizu and Imae, 1981), stress was evaluated using the Perceived Stress Scale (PSS) [Cohen et al. (1983); Japanese version: Sumi (2006)], and impulsivity was assessed using the Barratt Impulsivity Scale 11th version (BIS-11) (Patton et al., 1995; Japanese version: Kobashi and Ida, 2013).

## Computational Models

**Figure 3:**
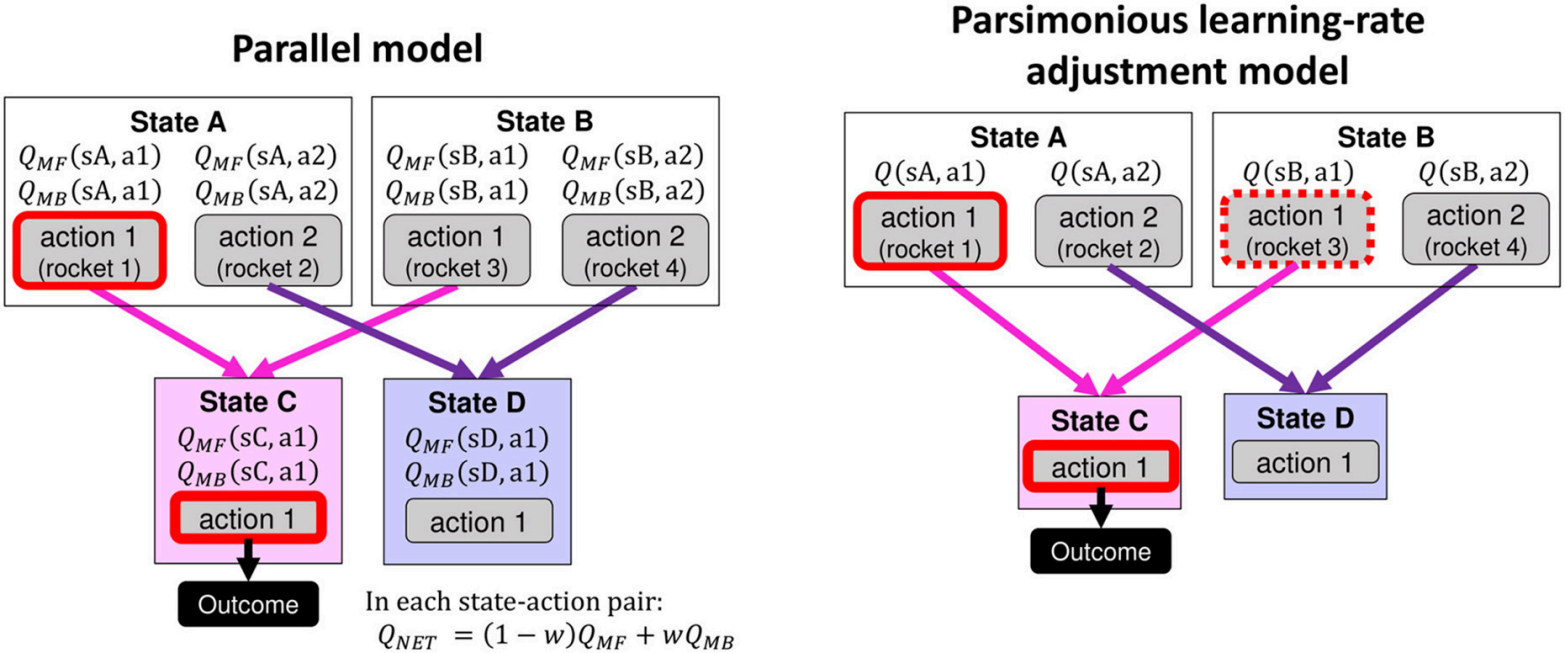
A schematic of value updating in the parallel model **(left panel)** and the parsimonious learning-rate adjustment model **(right panel)**. The panels show the difference in value updating in the two models when the agent selected action 1 in state A followed by state C and an outcome. The parallel model assumes that model-free values are updated for the experienced state-action pairs and that model-based values are updated for every state-action pair. These values are mixed according to a model-based weighting parameter *w*. On the other hand, the parsimonious learning-rate adjustment model updates only the first-stage actions relating to the state that produced the outcome based on the transition-probability model. In the example shown in this figure, the value of action 1 in state A is updated by the outcome in state C. At the same time, the value of action 1 in state B is also updated but downweighted by the model-based weighting parameter *w*.

We first describe two basic models (the parallel model and the parsimonious learning-rate adjustment model, Figure 3) as candidates to explain the data for the two-step task. In addition, we introduce some variations of the forgetting process that can be combined with these models.

### Parallel Model (Original Model)

For data from the Kool two-step task, a computational model developed by Daw et al. (2011) is ordinarily used. This model uses the *forward-looking* model-based system and assumes that the model-free and model-based values are computed in parallel and combined as a net value for a choice weighted by the weighting parameter *w* (Figure 3, left). Hereafter, we call this model the *parallel model* (or P model).

#### Value Calculation

The model-free learning system uses a SARSA (λ) TD learning rule (Rummery and Niranjan, 1994) and updates state-action values, *Q*_*MF*_(*s*_*i, t*_, *a*_*i, t*_), at each stage *i* of each trial *t*. In the current task, two states are included in each stage (*s*_*A*_ and *s*_*B*_ for *s*_1,*t*_, and *s*_*C*_ and *s*_*D*_ for *s*_2,*t*_). In each of the first-stage states, two actions are available, and *a*_*i, t*_ ∈ *a*_1_, *a*_2_ denotes the selected action. In the second-stage state, only one action is available. In both stages, the selected state-action value is updated as follows:

(1) $$\begin{array}{l} Q_{MF}\left(s_{i,t},\;a_{i,t}\right)\leftarrow Q_{MF}\left(s_{i,t},\;a_{i,t}\right)\\ \;+\;\alpha_{L}\left(r_{i,t}+Q_{MF}\left(s_{i+1,t},\;a_{i+1,t}\right)-Q_{MF}\left(s_{i,t},\;a_{i,t}\right)\right), \end{array}$$

where 0 ≤ α_*L*_ ≤ 1 is the learning rate parameter and 0 ≤ *r*_*i,t*_ ≤ 1 denotes the reward in trial *t*, which is linearly transformed from actual feedback ranging from 1 to 9. Specifically, the selected first- and second-stage values, respectively, are updated as follows:

(2) $$\begin{array}{l} Q_{MF}\left(s_{1,t},\;a_{1,t}\right)\leftarrow Q_{MF}\left(s_{1,t},\;a_{1,t}\right)\\ \;+\;\alpha_{L}\left(Q_{MF}\left(s_{2,t},\;a_{2,t}\right)-Q_{MF}\left(s_{1,t},\;a_{1,t}\right)\right),\; \end{array}$$

(3) $$\begin{array}{l} Q_{MF}\left(s_{2,t},\;a_{2,t}\right)\leftarrow Q_{MF}\left(s_{2,t},\;a_{2,t}\right)\\ \;+\;\alpha_{L}\left(r_{2,t}-Q_{MF}\left(s_{2,t},\;a_{2,t}\right)\right). \end{array}$$

The second-stage reward prediction error (RPE), which reflects the difference between the expected and actual reward, also updates the first-stage value but is downweighted by the eligibility trace decay parameter λ as follows:

(4) $$\begin{array}{l} Q_{MF}\left(s_{1,t},\;a_{1,t}\right)\leftarrow Q_{MF}\left(s_{1,t},\;a_{1,t}\right)\\ \;+\;\alpha_{L}\lambda \left(r_{2,t}-\;Q_{MF}\left(s_{2,t},\;a_{2,t}\right)\right), \end{array}$$

where λ denotes the trace decay parameter that modulates the magnitude of the effect of the second-stage RPE on the first-stage value. This type of updating is called the eligibility trace rule and enables efficient value updating (Sutton and Barto, 1998).

The model-based values, *Q*_*MB*_, for each action are defined by the Bellman optimality equation. In short, an option value is computed anew each time as a sum of the maximum values of the possible subsequent state-action values weighted by the transition probabilities for the respective states. The transition probability determines this weight. Thus, model-based values *Q*_*MB*_(*s*_*j*_, *a*_*k*_), where *s*_*j*_ ∈ *s*_*A*_, *s*_*B*_, *s*_*C*_, *s*_*D*_, and *a*_*k*_ ∈ *a*_1_, *a*_2_ in the first stage and *a*_*k*_ = *a*_1_ in the second stage, are calculated as follows:

(5) $$Q_{MB}\left(s_{j},a_{k}\right)=\sum_{s^{\prime }}T(s^{\prime }|s_{j},a_{k})\underset{a}{\text{max}}\;Q_{MB}(s^{\prime },a).$$

Here, *T*(*ś*|*s*_*j*_, *a*_*k*_) is a transition-probability function representing the probability of moving to state *ś* after choosing action *a*_*k*_ at state *s*_*j*_. ***a*** represents possible actions at state *ś*, and the max operator indicates the maximum of all action values in state *ś*. In the Kool two-step task, the transition probability from the first-stage action to the second-stage state is determinate, and the second-stage state simply requires the subject to press the space bar. Thus, the first-stage model-based values are equal to the model-based value in state *ś* because *T*(*ś*|*s*_*j*_, *a*_*k*_) = 1 when *j* is *A* or *B*. Regarding the second stage (i.e., when *j* is *C* or *D*), model-based values are equivalent to model-free values because no transition to a further stage occurs.

Finally, *Q*_*MF*_ and *Q*_*MB*_ are integrated to generate a net value for choice with a model-based parameter 0 ≤ *w* ≤ 1 (Daw et al., 2011):

(6) $$\begin{array}{l} Q_{NET}\left(s_{j},\;a_{k}\right)=wQ_{MB}\left(s_{j},\;a_{k}\right)+{\left(1-w\right)Q}_{MF}\left(s_{j},\;a_{k}\right). \end{array}$$

The second-stage *Q*_*NET*_ values are equal to *Q*_*MB*_ and *Q*_*MF*_.

#### Choice Bias

These net values determine the first-stage choice probability of choosing action *a* among the candidate actions, *P*(*a*_1,*t*_ = *a*|*s*_1,*t*_), as follows:

(7) $$\begin{array}{l} P\left(a_{1,t}=a|s_{1,t}\right)\\ =\;\frac{\exp\left[\beta \cdot Q_{NET}\left(s_{1,t},a\right)+\pi \cdot rep\left(s_{1,t},a\right)+\rho \cdot resp\left(s_{1,t},a\right)\right]}{\sum_{á}\exp\left[\beta \cdot Q_{NET}\left(s_{1,t},á\right)+\pi \cdot rep\left(s_{1,t},á\right)+\rho \cdot resp\left(s_{1,t},á\right)\right]}. \end{array}$$

Here, three free parameters represent particular propensities in the choice process: β, often called inverse temperature, adjusts how sharply the value difference between options is reflected in the choice probability; π determines the degree of perseveration in the same option; and ρ expresses the degree of key-response stickiness. *rep*(*a*) is an indicator variable that equals one if *a* is a first-stage action and is the same as the action chosen in the previous trial and zero otherwise. *resp*(*a*) is an indicator variable that equals one if *a* is a first-stage action using the same response key pressed in the previous trial and zero otherwise. Thus, the parameters π and ρ express perseveration (when the values are positive) or switching (when the values are negative) in favor of one option or one side, respectively. If β = 0, then calculated value differences have no influence on choice probabilities, and if β → ∞, then the maximum-value option is always chosen.

Among these parameters, β is usually included in any RL model. In the two-step tasks, Daw et al. (2011) used π but did not use ρ, whereas Kool et al. (2016) used both π and ρ. We examined the models lacking π, ρ, or both, but they were not supported by model selection; therefore, in the Results section, we will report the models using both π and ρ as free parameters.

### Parsimonious Learning-Rate Adjustment Model

As another framework, we propose a parsimonious computational model applying cognitive savings of the values to be updated (Figure 3, right). Under this framework, a deterministic action sequence after one makes a choice is regarded as a unit for valuation, and only the action values in the choice stage are computed. In the current task, the choice stage corresponds to the first stage. This framework realizes computational savings and one practical solution for the problem of temporal granularity in TD learning. In this framework, where only the first-stage values are updated, the forward-looking model-based system cannot calculate the model-based values because it needs the future state values (see Equation 5). Therefore, we applied the *backward-looking* model-based system in which the degree of value updating by outcome is adjusted in a model-based manner (Figure 2B, right). When there are multiple sets of choices before the outcome feedback, the eligibility trace decay parameter is adjusted in a model-based manner (Toyama et al., 2017), but when there is only one choice, as in the case of the Kool two-step task, the system can be implemented by adjusting the learning rate parameter. Thus, we refer to this model as the *parsimonious learning-rate adjustment model* (the parsimonious LA model, hereafter called the “LA model” for brevity). This model has another parsimonious aspect: it uses the backward-looking model-based system, which assumes only one value for one action (in contrast, the forward-looking model-based system calculates model-free and model-based values in parallel and combines them into a net value for each action).

#### Value Calculation

In this model, a deterministic action sequence followed by a choice is the unit for valuation, and only the action values in the choice stage are updated. In the Kool two-step task, these values correspond to the values of the first-stage rockets, *Q*(*s*_*A*_, *a*_1_), *Q*(*s*_*A*_, *a*_2_), *Q*(*s*_*B*_, *a*_1_) and *Q*(*s*_*B*_, *a*_2_). Here, choosing *a*_1_ deterministically leads to *s*_*C*_, and choosing *a*_2_ leads to *s*_*D*_. We use *Q* in this model because one value for one action is assumed. The first actions of the deterministic state-action sequences are updated as follows:

(8) $$\begin{array}{l} Q\left(s_{1,t},\;a_{1,t}\right)\leftarrow Q\left(s_{1,t},\;a_{1,t}\right)+\alpha_{L}\left(r_{2,t}-Q\left(s_{1,t},\;a_{1,t}\right)\right). \end{array}$$

The pure model-free value calculation ends here. If the backward-looking model-based system works, then the other state-action pair that leads to the same second-stage state with *a*_1,*t*_ is also updated as follows:

(9) $$\begin{array}{l} Q\left(\bar{s_{1,t}},\;a_{1,t}\right)\leftarrow Q\left(\bar{s_{1,t}},\;a_{1,t}\right)+w\alpha_{L}\left(r_{2,t}-Q\left(\bar{s_{1,t}},\;a_{1,t}\right)\right). \end{array}$$

Here, the weight of model-based updating is adjusted by a model-based parameter 0 ≤ *w* ≤ 1. The pure model-based system (*w* = 1) equally updates all the actions eligible for the outcome. For example, if an agent receives a certain amount of reward after choosing rocket 1, then he can speculate that the same reward would have been obtained if he had chosen rocket 3. Thus, he updates the value of rocket 3 in the same manner as rocket 1.

The LA model obviously has simpler calculations than the P model.

#### Choice Bias

This process is identical to that introduced in the P model (Equation 7).

### Forgetting Process

The values of unselected actions (including the actions of the unvisited state) are not updated in typical RL. However, these values can naturally be considered to decay through a forgetting process. The following equation is one algorithm for this process: the values of unselected actions are updated as follows in each step when a selected action is updated by Equations 2, 3 in the parallel model and by Equation 8 in the LA model:

(10) $$\begin{array}{l} Q_{MF}\left({\tilde{s}}_{i,t},{ã}_{i,t}\right)\leftarrow Q_{MF}\left({\tilde{s}}_{i,t},{ã}_{i,t}\right)+\alpha_{F}\left(\mu -Q_{MF}\left({\tilde{s}}_{i,t},{ã}_{i,t}\right)\right),\; \end{array}$$

where 0 ≤ α_*F*_ ≤ 1 is the forgetting rate parameter and 0 ≤ μ ≤ 1 is the default-value parameter to which the values of unselected options are regressed. $Q_{MF}\left({\tilde{s}}_{i,t},{ã}_{i,t}\right)$ represents the unselected and unvisited state-action values in stage *i* at trial *t* (such that $\left({\tilde{s}}_{i,t},{ã}_{i,t}\right)\neq \left(s_{i,t},a_{i,t}\right)\;for\;each\;i$). As a predicted tendency, a small μ promotes the avoidance of unselected options, whereas a large μ promotes a propensity for unselected options. Strictly, this tendency is determined by comparison with the recently chosen option value. This forgetting algorithm was first examined for a multistep decision-making task in our previous paper (Toyama et al., 2017) and improved the model fit for the data from the Daw two-step task, but they are still not widely used. Additionally, the current study examines a model without a forgetting process (where α_*F*_ = 0) and three types of models with a forgetting process: the first model assumes that the values of unselected options gradually approach zero (where α_*F*_ is a free parameter and μ = 0), the second model assumes that they approach 0.5, which corresponds to the least biased value (where α_*F*_ is a free parameter and μ = 0.5), and the third model assumes that people have their own default value to which the values approach (where both α_*F*_ and μ are free parameters).

## Measures of Model Fitting and Selection Criteria

We used the R function “solnp” in the Rsolnp package (Ghalanos and Theuss, 2015) to estimate the free parameters. For a comparison of these models, we computed the Akaike information criterion [AIC; Akaike (1974)], which is given by

(11) $$\begin{array}{l} AIC=-2LL+2k, \end{array}$$

where *LL* is the log likelihood and *k* is the number of free parameters. The model with a smaller value is considered the preferred model. We used this criterion to compare the predictive ability of the models.

## Results

### Overall Model Comparison and Estimated Parameters

The negative LL and AIC of each participant were calculated for each model and were summed over all participants (*n* = 29) (Table 1). These models differ in the combination of *the basic model* (the parallel model or the LA model) and *the forgetting process* (no forgetting, forgetting in which μ = 0, forgetting in which μ = 0.5, or forgetting in which μ is a free parameter) and were denoted as the P, P-F0, P-F05, P-FD, LA, LA-F0, LA-F05, and LA-FD models. Table 1 also includes the results of the SARSA (λ) TD model that uses the pure model-free system and does not include the forgetting process, which is equivalent to the parallel model in which *w* = 0 for comparison. Among the models, the best-fitting model was the LA-F05 model, which showed the smallest AIC, followed by the LA-FD model. To examine the total improvement by the best-fitting model (the LA-F05 model) compared with the ordinarily used parallel model (the P model), we subtracted the AIC of the LA-F05 model from that of the P model for each participant. This calculation showed that the data for 28 of the 29 participants supported the LA-F05 model. In sections Overall model comparison and estimated parameters and Model differences and the estimated weighting parameters, additional comparisons are performed to determine whether the fitting improvement was attributed to the backward-looking model-based system of the LA models, forgetting process, or both.

**Table 1:** Information concerning the models compared on the basis of their fit to the choices of 29 participants.

**Model**	**Basic model**	**Forgetting**	**Default value**	**Free parameters**	**#**	**–LL**	**AIC**
SARSA (λ) TD	Model-free	–	–	α_*L*_, β, π, ρ, λ	5	4,661	9,612
P	Parallel	–	–	α_*L*_, β, π, ρ, *w*, λ	6	3,435	7,219
P-F0	Parallel	o	Fixed (μ = 0)	α_*L*_, β, π, ρ, *w*, λ, α_*F*_	7	3,284	6,974
P-F05	Parallel	o	Fixed (μ = 0.5)	α_*L*_, β, π, ρ, *w*, λ, α_*F*_	7	3,048	6,503
P-FD	Parallel	o	o	α_*L*_, β, π, ρ, *w*, λ, α_*F*_, μ	8	3,024	6,511
LA	Learning-rate adjustment	–	–	α_*L*_, β, π, ρ, *w*	5	3,447	7,184
LA-F0	Learning-rate adjustment	o	Fixed (μ = 0)	α_*L*_, β, π, ρ, *w*, α_*F*_	6	3,292	6,931
LA-F05	Learning-rate adjustment	o	Fixed (μ = 0.5)	α_*L*_, β, π, ρ, *w*, α_*F*_	6	3,055	6,457
LA-FD	Learning-rate adjustment	o	o	α_*L*_, β, π, ρ, *w*, α_*F*_, μ	7	3,032	6,469

*This list provides information for the free parameters, negative log likelihood (–LL) and Akaike information criterion (AIC) summed over all participants (n = 29) for each model. The models differ regarding the basic model with parallel (P) or parsimonious learning-rate adjustment (LA). The models also differ regarding their forgetting-process assumptions: no forgetting, forgetting with a fixed default value (F0 or F05), or forgetting with a free default value parameter (FD)*.

Table 2 shows the estimated parameters using the P, LA, P-F05, LA-F05, P-FD, LA-FD models for the comparison. Interestingly, for most of the participants, the estimated learning rates were extremely high. In addition, in the parallel models, the estimated λ values were also extremely high for most of the participants. Therefore, we examined additional parallel models in which α_*L*_ and λ were fixed at 1 and additional LA models in which α_*L*_ was fixed at 1. These reduced models showed lower AIC values (Table S2) compared with the models including the full parameters (hereafter called the full models). Interestingly, if α_*L*_ and λ are set to one, then the parallel models have a similar structure to the LA models in which α_*L*_ is set to one. In these models, the second-stage state values are equal to the last piece of feedback if α_*L*_ = 1, and the last piece of feedback is directly reflected in the first-stage value because λ = 1. Thus, such specific parallel models behave similarly to the LA models, which do not distinguish the first-stage state-action value and the following second-stage state-action value. Note that these results support the parsimonious updating assumed in the LA models but provide no information on the comparison between the forward-looking and the backward-looking model-based systems.

**Table 2:** Estimated parameter values.

**Model**	**Percentile (%)**	****α**_L_**	****β****	**w**	****π****	****ρ****	****λ****	****α**_F_**	****μ****
P	25	0.98	3.34	0.70	−0.45	−0.33	0.17	–	–
	50	**1.00**	**4.31**	**0.90**	–**0.16**	–**0.15**	**0.97**	–	–
	75	1.00	5.39	1.00	0.24	−0.04	1.00	–	–
LA	25	1.00	3.19	0.71	−0.42	−0.33	–	–	–
	50	**1.00**	**4.06**	**0.87**	–**0.15**	–**0.14**	–	–	–
	75	1.00	5.37	1.00	0.26	−0.05	–	–	–
P-F05	25	0.86	5.37	0.49	0.16	−0.31	0.88	0.21	Fixed (0.5)
	50	**1.00**	**7.59**	**0.67**	**0.40**	–**0.18**	**1.00**	**0.30**	**Fixed (0.5)**
	75	1.00	10.53	0.74	0.61	−0.06	1.00	0.49	Fixed (0.5)
LA-F05	25	0.88	5.69	0.43	0.21	−0.32	–	0.23	Fixed (0.5)
	50	**0.97**	**7.71**	**0.57**	**0.45**	–**0.17**	–	**0.30**	**Fixed (0.5)**
	75	1.00	10.43	0.76	0.62	−0.04	–	0.52	Fixed (0.5)
P-FD	25	0.84	5.24	0.51	0.16	−0.32	0.89	0.21	0.46
	50	**1.00**	**8.28**	**0.64**	**0.52**	–**0.17**	**1.00**	**0.28**	**0.53**
	75	1.00	10.09	0.75	0.95	−0.06	1.00	0.45	0.62
LA-FD	25	0.84	5.30	0.44	0.16	−0.32	–	0.23	0.47
	50	**0.91**	**8.43**	**0.58**	**0.63**	–**0.16**	–	**0.27**	**0.52**
	75	1.00	11.02	0.80	1.00	−0.06	–	0.47	0.62

*The best-fitting parameter values are shown as the median and 25th and 75th percentiles across participants. The models differ regarding the basic model with parallel (P) or parsimonious learning-rate adjustment (LA). The models also differ regarding their forgetting-process assumptions: no forgetting, forgetting with a fixed default value (F05), or forgetting with a free default value parameter (FD). Median values are written in bold*.

#### Effect of the Model-Based System

In the full models, the AIC values were lower in the LA models than those in the parallel models: The LA model was favored over the P model, the LA-F05 model was favored over the P-F05 model, and the LA-FD model was favored over the P-FD model (favored by more than 20 of 29 participants in each comparison; *p*s < 0.005 for the paired *t*-tests). However, in the reduced models, no significant differences in AIC values were observed between the basic models with any forgetting process assumptions (i.e., all *p*s > 0.10 in the paired *t*-tests).

Based on this result, the higher AIC values in the parallel models than those in the LA models among the full models are attributable to the effect of the redundant free parameters in the parallel models, and the difference in the model-based system (parallel or LA) is not critical for fitting improvement.

#### Effect of the Forgetting Processes

Regardless of the model comparisons among the full models or those among the reduced models, the models with forgetting processes were favored. Here, we show only the results of the full models, but the similar results were obtained for the reduced models (Supplementary Text 1.2).

**Figure 4:**
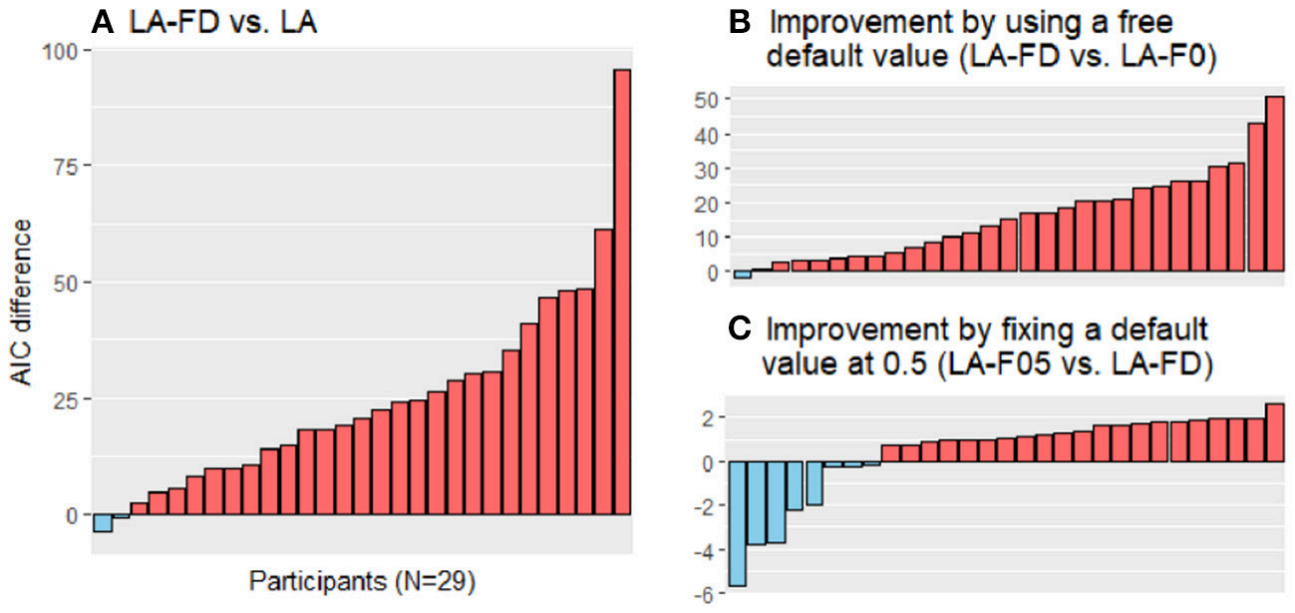
Model comparison by differences in the Akaike information criterion (AIC) scores in the parsimonious learning-rate adjustment models (LA models). The AIC scores of the LA models were compared. One of the models has no forgetting process (LA), and the other three have a forgetting rate parameter for the forgetting process and either a free default-value parameter (LA-FD), a fixed default value of 0 (LA-F0), or a default value of 0.5 (LA-F05). **(A)** The LA-FD model was favored over the LA model (red bars favor the LA-FD model, and blue bars favor the LA model). **(B)** By including a default-value parameter, the data fit was improved (red bars favor the LA-FD model, and blue bars favor the LA-F0 model). **(C)** No statistically significant improvement was observed by fixing the default value at 0.5 (red bars favor the LA-F05 model, and blue bars favor the LA-FD model). Note that the scales of the vertical axis are different among the panels.

**Figure 5:**
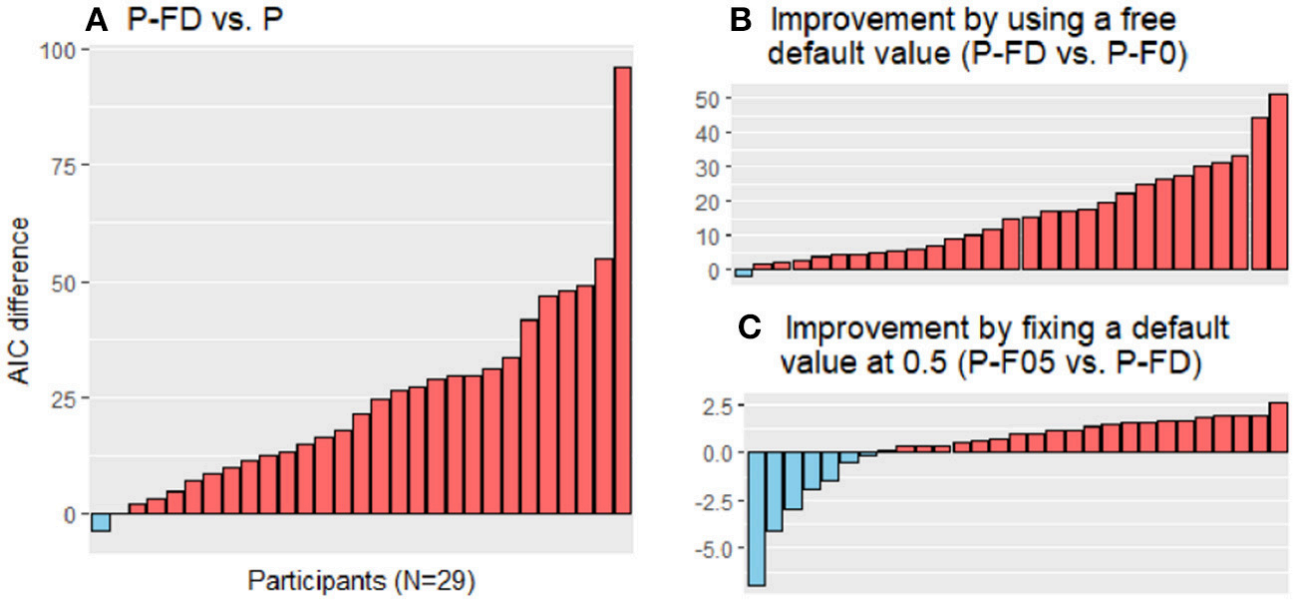
Model comparison by differences in the Akaike information criterion (AIC) scores in the parallel models (P models). The AIC scores of the P models were compared. One of the models has no forgetting process (P), and the other three have a forgetting rate parameter for the forgetting process and either of a free default-value parameter (P-FD), a fixed default value of 0 (P-F0), or a default value of 0.5 (P-F05). **(A)** The P-FD model was favored over the P model (red bars favor the P-FD model, and blue bars favor the P model). **(B)** By including a default-value parameter, the data fit was improved (red bars favor the P-FD model, and blue bars favor the P-F0 model). **(C)** No statistically significant improvement was observed by fixing the default value at 0.5 (red bars favor the P-F05 model, and blue bars favor the P-FD model). Note that the scales of the vertical axis are different among the panels.

Most participants showed reduced AIC values in the LA-FD model vs. the LA model [Figure 4A, favored by 27 of 29 participants, *t*_(28)_ = −6.30, *p* < 0.001] and in the P-FD model vs. the P model [Figure 5A, favored by 27 of 29 participants, *t*_(28)_ = −6.24, *p* < 0.001]. These results strongly suggest that the forgetting process cannot be neglected in constructing the framework of value-based learning.

Among the models with forgetting processes, assuming that the default value was a free parameter was preferred rather than assuming it was 0, with lower AIC values in the LA-FD model than those in the LA-F0 model by 28 of 29 participants [Figure 4B, *t*_(28)_ = −6.60, *p* < 0.001] and in the P-FD model than those in the P-F0 model by 28 of 29 participants [Figure 5B, *t*_(28)_ = −6.37, *p* < 0.001]. However, we did not find significant differences between the LA-F05 and LA-FD models (Figure 4C, *p* = 0.34) or between the P-F05 and P-FD models (Figure 5C, *p* = 0.28), although 20 participants favored the LA-F05 model over the LA-FD model and the P-F05 model over the P-FD model according to the AIC scores. These results may reflect the current task setting in which the average expected outcome over the task was close to 0.5 (when the points in the task were linearly transformed to the range of 0–1); the expected outcome was 0.46 under random choice and 0.53 on average among the participants.

### Model Differences and the Estimated Weighting Parameters

In the previous section, we reported that the model fits were improved by using the reduced models: the LA models in which α_*L*_ was fixed and the P models in which α_*L*_ and λ were fixed. To assess the influence of fixing these parameters on the estimation of the weighting parameter *w*, we conducted linear regression analyses and confirmed that the estimations of *w* were not different between the full models and the reduced models (Figure S2). Therefore, in the following analyses, we report the results of the full models.

**Figure 6:**
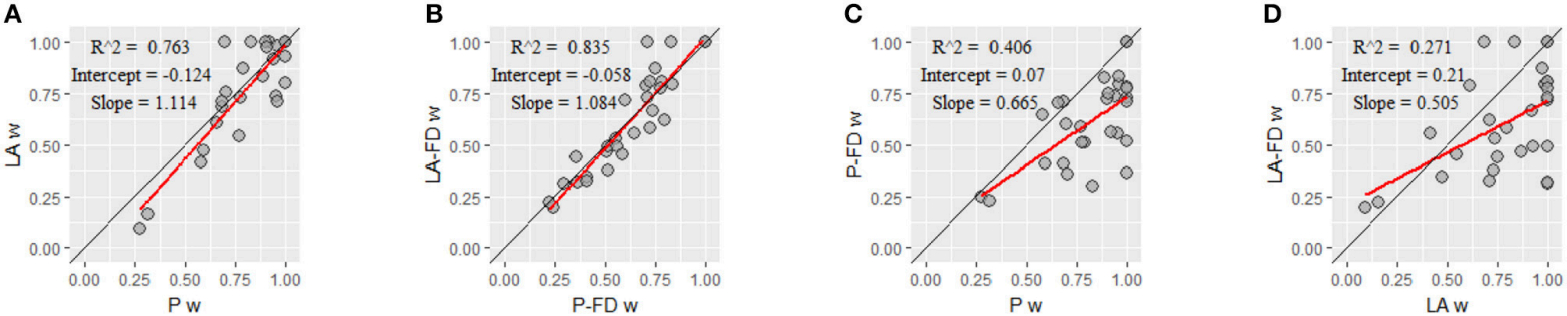
The correspondence of the estimated weighting parameter *w* by different models. Regarding the weighting parameter *w*, the correspondence **(A)** by the parallel model (P model) and the parsimonious learning-rate adjustment model (LA model), **(B)** by the P model with forgetting (P-FD model) and the LA model with forgetting (LA-FD model), **(C)** by the P model and the P-FD model, and **(D)** by the LA model and the LA-FD model are shown. Each panel shows the coefficient of determination (R^2^), regression line intercept, and regression line slope. Red lines indicate linear regression lines. The data on the black lines indicate complete correspondence between the estimations by the two models.

First, we examined differences in the basic models with respect to the estimations of *w*. Figure 6A shows the linear regression of the LA model on the P model (intercept = −0.1, slope = 1.1, *R*^2^ = 0.76), and Figure 6B shows the linear regression of the LA-FD model on the P-FD model (intercept = −0.1, slope = 1.1, *R*^2^ = 0.84). Some estimation differences emerged between the P models and the LA models, but the estimated regression slopes were close to 1. We also examined the influence of the forgetting processes on the estimations of the weighting parameter *w*. Figure 6C shows the linear regression of the P-FD model on the P model (intercept = 0.1, slope = 0.7, *R*^2^ = 0.41), and Figure 6D shows the linear regression of the LA-FD model on the LA model (intercept = 0.2, slope = 0.5, *R*^2^ = 0.27). The regression analyses revealed that the models with forgetting processes had lower estimated *w* values than those in the models without forgetting processes.

### Relationships Between Estimated Parameters and Data Characteristics or Scores on the Self-Reported Questionnaires

The analyses in this section were conducted to understand the characteristics of the model parameters. As observed previously, the P-F05 and LA-F05 models showed lower AIC values than the P-FD and LA-FD models, respectively, although no significant differences were noted. However, in this section, we mainly used the parameters estimated by the P-FD and LA-FD models to avoid possible estimation biases of *w* caused by fixing μ to 0.5 for some participants. We also provide the results of the analyses using the P and LA models for comparison.

#### Sensitivity to the Previous Outcome and the Weighting Parameter

The computational models were developed supposing that the weighting parameter *w* reflects use of the “model,” or the transition structure; therefore, we examined this assumption from the statistical characteristics of the obtained data. Considering that the Kool two-step task includes trials that prompt the use of the transition structure (MB trials) and trials that do not (MF trials), if a participant uses the “model,” he or she can be predicted to behave similarly in both types of trials, and this tendency is expected to be captured by the parameter *w*.

**Figure 7:**
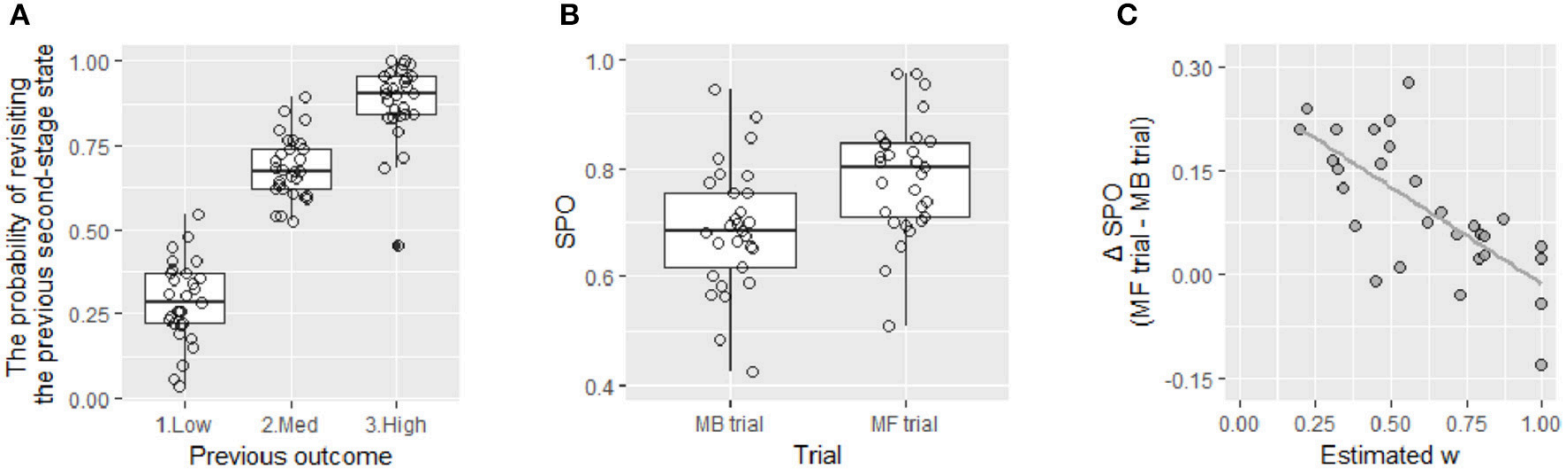
**(A)** This boxplot shows the average probabilities of revisiting the second-stage state that was visited in the previous trial given low (1~3 points), medium (4~6 point), and high (7~9 points) previous rewards. This probability obviously changed depending on the previous outcome. **(B)** This boxplot shows the average sensitivity to the previous outcomes (SPO) for choices in model-based (MB) trials and model-free (MF) trials. This sensitivity is calculated as the average ratio of the probability of revisiting the same second-stage state after a high reward to the same probability after a low reward. This score was significantly higher in MF trials than that in MB trials. **(C)** This panel shows the relationship between the parameter *w* estimated by the LA-FD model and the difference in SPO between the MF and MB trials (ΔSPO). A negative relationship was found between these variables, showing that participants with a low *w* were more sensitive to the previous reward in the MF trials vs. the MB trials.

To confirm this prediction, we focused on sensitivity to the outcome experienced in the previous trial. Generally, people revisit the state that recently produced high rewards. This pattern was also evident in our data. Figure 7A shows the probabilities of revisiting the same second-stage state for three previous outcome conditions, which are designated Low (1~3 points), Medium (4~6), and High (7~9). This probability was lowest in the Low condition (*M* = 0.28, *SE* = 0.02) and highest in the High condition (*M* = 0.88, *SE* = 0.02). Next, we calculated the ratio of the probabilities in the High and Low conditions, High/(High + Low), and defined this value as the index of *sensitivity to the previous outcome* (SPO). This index was significantly lower in the MB trials than that in the MF trials [Figure 7B, *t*_(28)_ = −5.32, *p* < 0.001) as expected because only the MB trials require use of the “model” to reach a certain second-stage state based on the previous outcome.

Those who can use the “model” should show a similar SPO in both the MF and MB trials, whereas those who cannot use the “model” should exhibit a higher SPO in the MF trials than that in the MB trials. Therefore, if the parameter *w* reflects use of the “model,” then a difference in the SPO (ΔSPO), which is defined by subtraction of the SPO in the MB trials from the SPO in the MF trials, can be predicted to negatively correlate with the parameter *w*. Then, we conducted Pearson correlation analyses between the estimated *w* and ΔSPO, as well as the SPO in the MF trials and the SPO in the MB trials. When using the *w* estimated by the LA-FD model, the parameter *w* showed a positive relationship with the SPO in the MB trials (*r* = 0.37, *p* = 0.048) but not with the SPO in the MF trials (*r* = −0.25, *p* = 0.19). Importantly, the ΔSPO showed a strong negative correlation with the parameter *w* (*r* = −0.72, *p* < 0.001, Figure 7C), indicating that participants with a high *w* behave the same in the MF and MB trials, whereas participants with a low *w* base their choices on the previous outcome less often in the MB trials than in the MF trials. Notably, such correlations were not observed with other parameters (all *r*s < 0.33). These results suggest that the model-based parameter *w* uniquely captures use of the “model,” or the transition structure.

As a reference, we conducted the same analyses for the *w* estimated using the other models. In any models, the estimated *w* had no significant relationships with the SPO in the MF trials (all *r*s < 0.30) but had moderate positive relationships with the SPO in the MB trials (P-F05: *r* = 0.42, *p* = 0.022; LA-F05: *r* = 0.46, *p* = 0.012; P-FD: *r* = 0.27, *p* = 0.16; LA: *r* = 0.44, *p* = 0.017; P: *r* = 0.42, *p* = 0.025). Regarding the relationships with ΔSPO, the models with forgetting processes showed relatively stronger negative correlations (P-F05: *r* = −0.74, *p* < 0.001; LA-F05: *r* = −0.77, *p* < 0.001; P-FD: *r* = −0.66, *p* < 0.001), although the models without forgetting showed no or weak negative correlations (LA: *r* = −0.33, *p* = 0.08; P: *r* = −0.38, *p* = 0.042). Taken together, the parameter *w* reflects the similarity of the magnitude of the effect of the latest outcome on choices in the MB trials to that in the MF trials, which is more clear when the models with forgetting processes are used.

#### Relationship With Total Reward

We next examined which parameter correlates with the total value of rewards obtained. Kool et al. (2016) reported that the model-based parameter *w* correlated with the total obtained reward. Contrary to this expectation, we did not find any relationship between *w* and total rewards (P: *r* = 0.24, *p* = 0.21; LA: *r* = 0.25, *p* = 0.19; P-FD: *r* = −0.15, *p* = 0.42; LA-FD: *r* = −0.11, *p* = 0.57). This unexpected result may have occurred because the relationship was too weak to detect from the current small sample size. In fact, our simulation using 200 samples generated from the best-fit parameter sets revealed positive relationships between *w* and the average reward in all models (Figure S3).

In addition, many other factors may be related to total rewards other than *w*, such as the reward schedule of the second-stage states and the contributions of other parameters to agents' choices. In particular, we found a strong positive correlation between total rewards and the value-based parameter β (P: *r* = 0.82, *p* < 0.001; LA: *r* = 0.81, *p* < 0.001; P-FD: *r* = 0.77, *p* < 0.001; LA-FD: *r* = 0.78, *p* < 0.001). We also found a moderate positive correlation between total rewards and the forgetting rate α_*F*_ when using the models with forgetting processes (P-FD: *r* = 0.44, *p* = 0.017; LA-FD: *r* = 0.48, *p* = 0.008), which may reflect the nature of this task that the immediately preceding outcome is the most informative.

#### Relationships With Self-Reported Psychopathology or Other Traits

Previous studies have reported a negative association between the weighting parameter and psychopathology, especially obsessive-compulsivity (Voon et al., 2015; Gillan et al., 2016; Patzelt et al., 2018). Considering that the model structure is critical for parameter estimation, we examined the relationships between the estimated parameter values and scores on questionnaires regarding psychopathology and other traits using the P, LA, P-FD, and LA-FD models (Table 3; the results of the reduced models are provided in Table S3 as a reference). The *w* estimated by the models with forgetting processes had no significant relationship with self-reported OCD tendencies as measured by the OCI contrary to our expectation (P-FD: *r* = −0.10, *p* = 0.60; LA-FD: *r* = −0.07, *p* = 0.71), whereas the *w* estimated by the models without forgetting processes showed weak negative correlations (P: *r* = −0.31, *p* = 0.098; LA: *r* = −0.36, *p* = 0.056).

**Table 3:** Associations of estimated parameter values with psychopathology and other traits.

	****α**_L_**	****β****	**w**	****π****	****ρ****	****λ****	****α**_F_**	****μ****
**Correlation with OCI**								
P	**−0.59**	**−0.42**	–*0.31*	**0.37**	–0.11	0.06		
LA	**−0.62**	**−0.40**	–*0.36*	**0.37**	–0.10			
P-FD	–0.24	**−0.39**	–0.10	–0.12	–0.11	0.06	–0.19	–0.06
LA-FD	–*0.31*	–*0.36*	–0.07	–0.13	–0.11		–0.21	–0.04
**Correlation with STAI**								
P	–*0.34*	0.02	0.08	–0.09	0.04	–0.21		
LA	**−0.38**	0.03	0.11	–0.11	0.02			
P-FD	–0.01	0.12	0.07	–0.03	–0.04	0.11	*0.32*	0.02
LA-FD	–0.12	0.15	0.05	–0.03	–0.03		0.29	0.08
**Correlation with SDS**								
P	–0.08	–0.01	0.15	–0.04	–0.10	–*0.32*		
LA	–0.06	0.00	0.15	–0.05	–0.11			
P-FD	0.20	–0.03	0.12	–0.10	–0.12	0.09	**0.41**	0.02
LA-FD	0.06	0.00	0.14	–0.07	–0.11		**0.41**	0.09
**Correlation with PSS.10**								
P	–0.07	0.06	0.23	–0.27	–0.14	**−0.44**		
LA	–0.07	0.06	0.18	–0.27	–0.13			
P-FD	0.08	0.10	0.25	–*0.34*	–0.18	–0.10	**0.39**	–0.06
LA-FD	–0.09	0.14	*0.31*	–*0.36*	–0.16		**0.39**	–0.05
**Correlation with BIS11**								
P	–0.09	–0.20	0.29	0.10	–0.18	–*0.36*		
LA	–0.10	–0.19	0.25	0.10	–0.18			
P-FD	0.07	–0.09	**0.54**	0.01	–0.25	–0.01	–0.10	–0.03
LA-FD	–0.04	–0.07	**0.56**	–0.02	–0.23		–0.12	–0.05

*The questionnaires used in this study were the Obsessive-Compulsive Inventory (OCI) for obsessive-compulsive disorder, the trait portion of the State-Trait Anxiety Inventory (STAI) for trait anxiety, the Self-Rating Depression Scale (SDS) for depression, the Perceived Stress Scale (PSS) for stress, and the Barratt Impulsivity Scale 11th version (BIS-11) for impulsivity. The correlations of their scores with the model parameters are shown. Model parameters were estimated using the parallel model (P), the parsimonious learning-rate adjustment model (LA), the P model in which μ is a free parameter (P-FD), or the LA model in which μ is a free parameter (LA-FD). Italic: p < 0.10. Bold: p < 0.05*.

This change in the correlations can be explained as follows: (1) the reduction in the AIC values by using the P-FD or LA-FD model instead of the P or LA model showed a marginally significant negative correlation with OCI scores (*r* = −0.33, *p* = 0.079; *r* = −0.34, *p* = 0.072); (2) this negative correlation indicates that the effect of the forgetting process was greater in participants with low OCI scores than that in participants with high OCI scores; (3) the values of *w* estimated by the models with forgetting processes (the P-FD and LA-FD models) were lower than those estimated by the models without forgetting processes (the P or LA models); (4) therefore, the *w* of participants with low OCI scores decreased more than that of participants with high OCI scores, causing the negative correlations between *w* and OCI scores to nearly disappear.

When the models with a forgetting process were used for model fitting, the weighting parameter *w* showed a moderate positive relationship with impulsivity as measured by the BIS 11 (P-FD: *r* = 0.54, *p* = 0.003; LA-FD: *r* = 0.56, *p* = 0.001), which was surprising because impulsivity is generally considered negatively correlated with model-based behavior. A possible explanation for this result may be related to the role of *w* in this task; that is, a greater *w* can heighten the effect of the previous outcome, especially in the MB trials. Therefore, in this study using healthy university students, higher impulsivity may have heightened the effect of the last outcome on the current choice, which may have been reflected by a greater *w*. Returning to the relationship with OCD, other parameters showed some correlations with OCI scores (negative correlation: α_*L*_ *and β*; *positive correlation* : π).

The current correlation results may not be generalizable because of the small number of participants and the restricted population. However, the results showed how the differences between the computational models greatly affect the parameter estimates and their relationships with other indices.

## Discussion

We compared several computational models for data from a two-step task with a deterministic transition structure (Kool et al., 2016). Based on model comparisons and parameter estimations, the participants appeared to use a parsimonious computational algorithm. That is, reward feedback seemed to directly update the first action of the deterministic state-action sequences. In addition, model fits were improved by including forgetting processes, which update unselected option values. In the model with a forgetting process, the weighting parameter *w* strongly corresponded to the statistically characterized degree of “model” use, i.e., use of the transition structure. In addition, we showed the possibility that the models with forgetting processes result in different conclusions compared to the original model in terms of the relationships between the model parameters and psychopathology.

### Parsimonious Value Computation

Model comparisons and estimated parameters supported a learning process including parsimonious value computation, which assumes that values are updated for deterministic state-action sequences but not for every state-action pair. Such computational savings are useful in a real environment because computing every action value would require too many resources. Consider buying a canned coffee from a vending machine. You first decide which coffee you will buy, insert a coin, push the appropriate button, take the coffee out of the bottom box, open it, and taste it. These processes can be divided infinitely, but if the taste of the obtained coffee is not good, you will reevaluate only the first choice. An action sequence becomes automatic or habitual with repetition, and until the sequence is interrupted, individual actions do not need to be evaluated. Therefore, in the task with the deterministic transition structure, the deterministic action sequence can be regarded as a unit for value computation, and this view is supported by our results. Previous studies have also shown that an action sequence is used in the learning process (Dezfouli and Balleine, 2013; Dezfouli et al., 2014).

**Figure 8:**
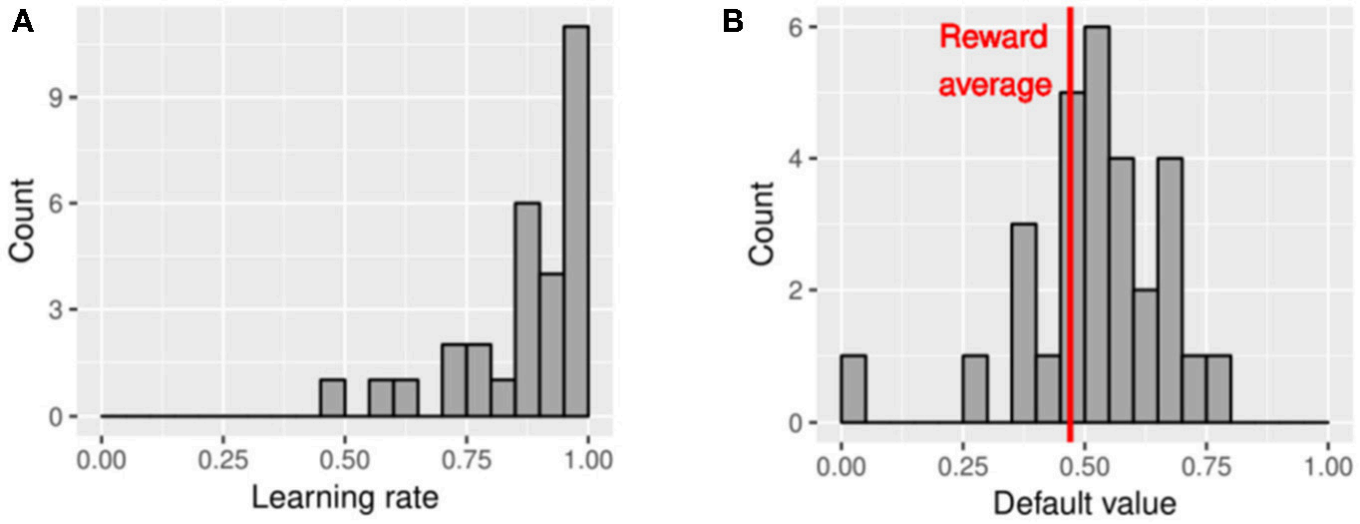
Histogram of the estimated parameter values of 29 participants. **(A)** Histogram of estimated learning rates. **(B)** Histogram of estimated default values. The reward average in this task was 4.7 points (equal to 0.46 in the computational model).

Interestingly, parameter estimation revealed that the choices in the current task were based on a more heuristic learning process; that is, the participants seemed to have recorded only the last outcome of each choice, which was presumed from our finding that the estimated learning rate (α_*L*_) was almost 1 for most of the participants (Figure 8A). These estimates were higher than those reported by Kool et al. (2017): median α_*L*_ = 0.82). Multiple potential reasons may explain the high learning rates in our data. First, the participants had sufficient time to be affected by the last outcome because the time limit for making a choice was longer than the periods that are ordinarily used [e.g., Kool et al. (2017) used a 1.5-s time limit]. Second, based on the nature of this task, the best method to predict the next option values is to record only the information from the immediately preceding outcome, or setting the learning rate to 1, because the outcomes change randomly and slowly every trial; most of the participants may have used this type of strategy. This heuristic learning process was revealed by fitting the data with the computational model. Obtaining this finding only from the statistical descriptions of the data is difficult.

### Forgetting Process

In our previous study (Toyama et al., 2017), we showed that the forgetting process, which assumes memory decay for unselected actions, improved the fit of the computational model for the data from the Daw two-step task. This result was replicated in the current study using the Kool two-step task. Because memory capacity is limited, retaining all action values in a stable manner is difficult. The values become noisy over time. Thus, the inclusion of forgetting processes may be reasonable to express natural choice behavior by the RL model. The standard RL model does not assume this process possibly because it was first developed in the field of engineering and may not need to assume that memory decay occurs.

In the current study, the models with the default value fixed at 0.5 showed the lowest AIC. Considering that expected outcome was 0.46 under random choice in our task, fixing the default value at 0.5 for all participants was reasonable, although the models including the default value as a free parameter also showed good model data fits, and variance in μ was observed among the participants (Figure 8B). The value of μ influences the likelihood of choosing the recently unselected options (Toyama et al., 2017). This tendency can be distinguished from a mere increase in choice randomness because randomness is expressed by the inverse temperature parameter (β*)*. Instead, μ expresses the value-based expectation assigned to the recently unselected options. Thus, if we use the model without a forgetting process, then choice shifts to unselected options are all erroneously expressed, for example, by decreasing β and the perseveration parameter (π). Thus, proper model construction is important to reduce the erroneous bias of the parameters.

Situations in which the forgetting process can affect the learning process are easy to conceptualize. For example, cognition regarding the task condition can affect the forgetting process. In a situation where the reward outcomes change frequently, the expectation for unselected options also becomes uncertain quickly, and the agent may change options often (expressed as a high forgetting rate). On the other hand, in a situation where the reward outcomes are stable, the expectation for unselected options is also stable (expressed as a low forgetting rate). Individual trait differences can also affect the forgetting process. For example, the difference between optimistic and pessimistic outlooks may be expressed as an individual difference in default values. Thus, the computational model with a forgetting process is expected to provide new insights in research related to value-based decision-making.

### Model-Based System

In this study, we could not determine which type of model-based system was used: the forward-looking or the backward-looking model-based system. This ambiguity emerged because a specific situation occurred (i.e., the estimated α_*L*_ and λ were almost one), implying that the current task was not appropriate to clarify which type of model-based system was used, and future studies using a proper task design that reflects the advantages and disadvantages of the two model-based systems are required. However, based on our previous work (Toyama et al., 2017) and this study, the backward-looking model-based system may work as well as the forward-looking system or more efficiently.

### Model-Based Parameter

The interpretation of the weighting parameter *w* is sometimes ambiguous in terms of whether it reflects the degree of learning about the transition model or the use of the transition model. The Kool two-step task has the advantage of restricting the parameter's meaning. Because this task has a clear deterministic transition structure, *w* is expected to mostly reflect the difference in the degree of model use among participants. In fact, we demonstrated the correspondence between the estimated *w* and individual differences in the model-free and model-based sensitivity to the previous outcome. This correspondence was found even when the parameter was estimated by the original parallel model, but the correlation was weaker than that when the parameter was estimated by the models with forgetting processes.

### Use of the Model Parameters as Indices

Previous studies have repeatedly found reduced use of the model-based system, which is defined by a low weighting parameter value, in OCD patients (Voon et al., 2015) and in participants with high OCD-like symptom severity as measured by self-reported questionnaires in a large-scale online study (Gillan et al., 2016; Patzelt et al., 2018). This stable predictability is a critical advantage of the parallel model. Our study also moderately replicated this finding when the parallel model was used, although only a marginally significant negative correlation was observed, possibly because of the small sample size. For example, Gillan et al. (2016) estimated that more than 1,000 participants were needed in their study using the Daw two-step task to achieve statistically significant results.

On the other hand, this correlation almost disappeared when the parameter *w* was estimated by the models with forgetting processes, implying that we must be careful when interpreting this parameter. First, considering that the model fits were improved for most of the participants by assuming that forgetting occurs, their choice data included a property corresponding to the forgetting process. Then, if the data are estimated by the models without forgetting, these models are forced to reflect this property only by their parameters. Our result showed that the values of *w* were estimated in a more model-based direction compared with the values estimated by the models with forgetting processes. The value changes affected the correlation between OCI scores and *w*, which may reflect the varying effect of the forgetting process among participants. Thus, the models with forgetting processes seem to lose their predictive ability for OCD tendencies, or at least their interpretability for OCD became complicated. OCD may require interpretation with a combination of parameters.

When using the models with forgetting processes, we found positive relationships between the weighting parameter *w* and impulsivity and between the forgetting rate α_*F*_ and depression or stress. Of course, considering the small sample size of this study, future studies are required to assess whether these models can provide useful predictive parameters. For example, the models must be applied to a large dataset for correlation analyses with clinical metrics and cognitive function. The model parameters must also be confirmed to be sufficiently recovered using various simulated behaviors (Palminteri et al., 2017). Ideally, the crucial parameters should be able to be appropriately estimated even when behaviors have additional or fewer characteristics than assumed in the models. In addition, regardless of differences in the model, task setting is also important to improve the sensitivity of the weighting parameter to individual differences. In the current task, more than twice as many MF trials as MB trials were included. By increasing the ratio of MB trials in future studies, the relationships between *w* and other individual traits may be clearer.

Although some future challenges remain, model fitting was notably improved for most of the participants by assuming a forgetting process, and as a result, the relationships between the parameters and self-reported psychopathology changed. If a data characteristic cannot be captured by a model, the model still must express the characteristic using its model parameters, which sometimes leads to misinterpretation of the parameters (Katahira, 2018). Therefore, the models with forgetting processes should be examined as candidate models to understand our cognitive process in future studies.

## Conclusion

The current study showed that participants favored the models with parsimonious computation, which assumes that the values are updated for action sequences, and a forgetting process, which assumes memory decay for unselected option values. Additionally, we confirmed that the estimated model-based weighting parameter could capture individual differences in “model use.” To date, however, most learning models do not contain psychological aspects such as cognitive savings and memory decay. Thus, research using the proposed model will force re-evaluation of how the features of the learning process correlate with psychopathology or abnormal decision-making and will enrich the study of the theory and neural basis of learning processes.

## Ethics Statement

This study was carried out in accordance with the recommendations of the ethical committee of Nagoya University with written informed consent from all participants. All participants gave written informed consent in accordance with the Declaration of Helsinki. The protocol was approved by the ethical committee of Nagoya University. The participants were all Nagoya university healthy students.

## Author Contributions

AT collected and analyzed the data and prepared the draft. KK and HO reviewed the manuscript critically and provided important intellectual input. All authors contributed to the design of the work as well as the interpretation of the results.

### Conflict of Interest Statement

The authors declare that the research was conducted in the absence of any commercial or financial relationships that could be construed as a potential conflict of interest.

## Footnotes

^1^For example, participants make two successive choices in the Daw two-step task but only one choice in the Kool two-step task. There are four options with different reward outcomes in the second stage of the Daw two-step task and two options with different reward outcomes in the second stage of the Kool two-step task. In addition, the current condition of each option is easy to assess in the Kool task using gradual integral point feedback, whereas the Daw two-step task uses binary feedback based on hidden probability.

^2^For example, considering that the final stage in the Daw two-step task has multiple options, the participants may intend to visit the same final state after they are not rewarded in that state because they can try the option that they did not choose in the previous trial. However, this strategy is not included in the existing computational models; thus, this choice behavior is sometimes regarded as a model-free strategy.
