# Supplementary material for: Reinforcement Learning With Parsimonious Computation and a Forgetting Process
Source: Front Hum Neurosci. 2019 May 9;13:153. doi: 10.3389/fnhum.2019.00153 (PMC6520826; doi:10.3389/fnhum.2019.00153)
Supplement: Supplementary file 1 [file Data_Sheet_1.docx]

Supplementary Material

Reinforcement learning with parsimonious computation and a forgetting process

Asako Toyama*, Kentaro Katahira, and Hideki Ohira

*** Correspondence:** asako.toyama@gmail.com

# Supplementary Text

## Task setting

Rewards have been reported to affect the model-based weight in the Kool two-step task (Kool et al., 2017). Therefore, we deliberately controlled the task conditions. First, preset rewards for states C and D in each trial were the same for all participants, as shown in Figure 1. In addition, as shown in Table S1, the average reward is almost the same between state C (mean = 4.68) and state D (mean = 4.72), and the average reward differences between the two states are almost the same when either state C (Table S1, mean=3.11) or state D is better (Table S1, mean = 3.24).

We prepared four trial orders that differed in the change points of the first-stage state (Figure S1, left). Each participant was allocated to one of the four sequences. In every sequence, the number of repetitions of the same state was 1, 2, 3, 4, 5, or 6, and each of these values was included 12 times before the last trial of the task. We also controlled the previous reward conditions between MF trials (180 trials) and MB trials (72 trials). The tables on the right-hand side of Figure S1 show the characteristics of the reward conditions.

## Effect of the forgetting processes in the reduced models

Regarding the effect of the forgetting processes in the reduced models, we reached the same conclusion as that for the full models. Most participants showed reduced AIC scores in the LA-FD model versus the LA model [favored by 26 of 29 participants, *t*(28) = -6.05, *p* < .001] and in the P-FD model versus the P model [favored by 26 of 29 participants, *t*(28) = -5.93, *p* < .001].

In the models with a forgetting process, assuming that the default value was a free parameter was preferred over assuming it was 0, indicating that the LA-FD model was favored over the LA-F0 model by 28 of 29 participants [*t*(28) = -6.34, *p* < .001] and that the P-FD model was favored over the P-F0 model by 28 of 29 participants [*t*(28) = -5.93, *p* < .001]. However, we did not find significant differences between the LA-F05 and LA models (*p* = .08) or between the P-F05 and P models (*p* = .06), although 21 and 23 participants favored the LA-F05 and P-F05 models according to the AIC, respectively.

# Supplementary Figures and Tables

## Supplementary figures


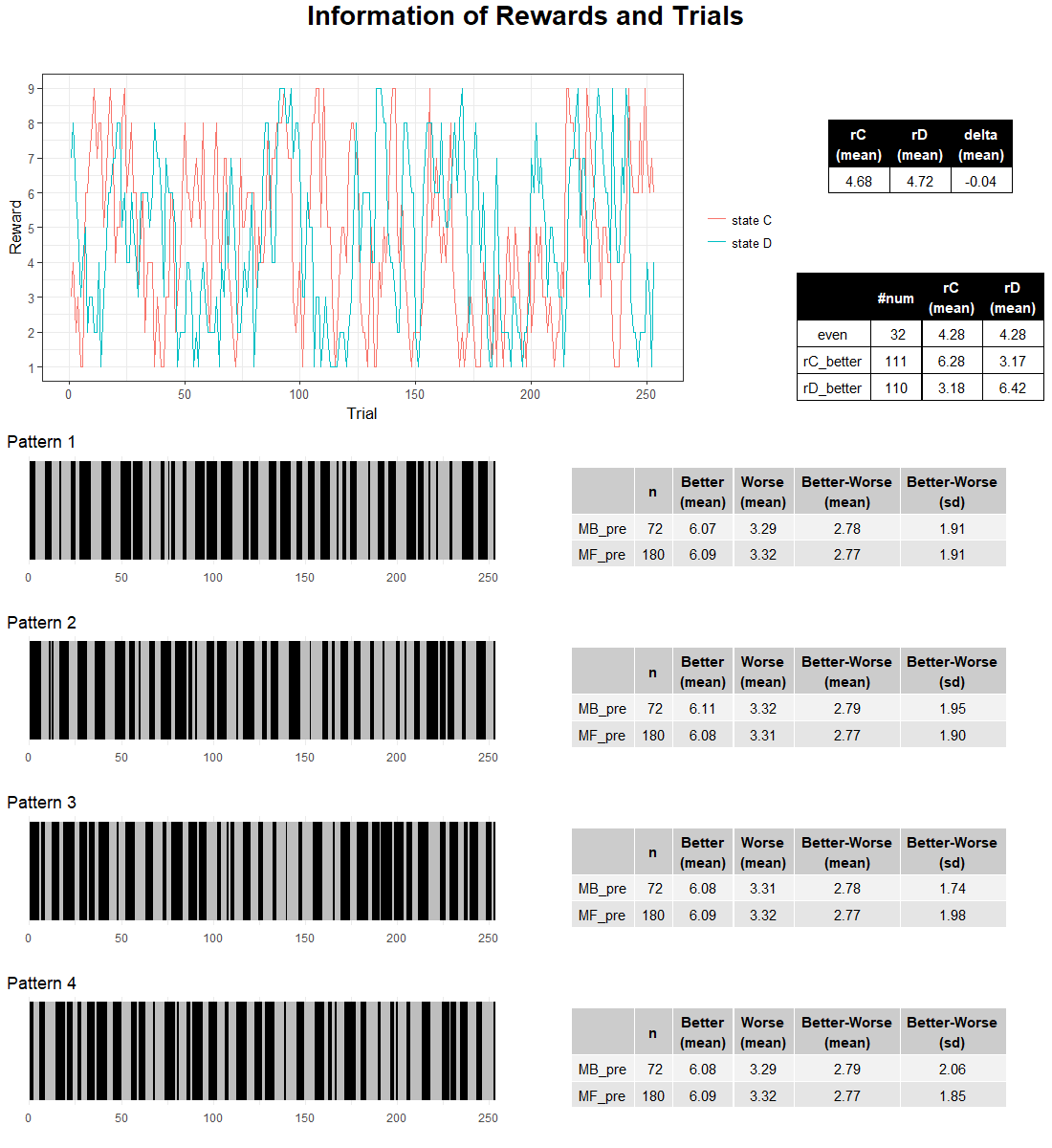


**Figure S1.** **The four trial orders used in the experiment.** The participants were allocated among the four trial orders. The figures on the left show which first-stage state was displayed in each trial during the task: the gray color indicates that the trial starts from state A, and the black color indicates that the trial starts form state B. The horizontal axis represents the trial number. The tables on the right show the reward characteristics of the trials immediately before the model-based trials (MB_pre) and immediately before the model-free trials (MF_pre). n is the number of trials excluding the first trial.

A. B. C. D.


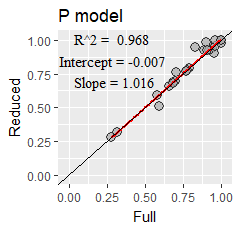

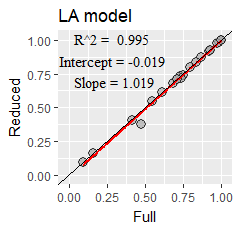

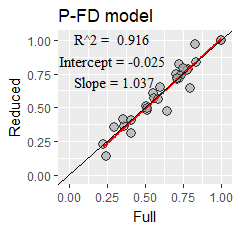

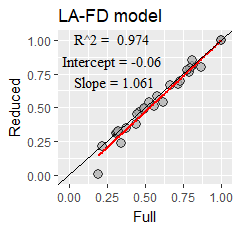


**Figure S2. The correspondence of the estimated weighting parameter *w* by the full and reduced models.** The correspondence of the estimated weighting parameter $w$ by the full and reduced models is shown: **(A)** the parallel model (P model), **(B)** the parsimonious learning-rate adjustment model (LA model), **(C)** the P model with forgetting in which $\mu$ is a free parameter (P-FD model), and **(D)** the LA model with forgetting in which $\mu$ is a free parameter (LA-FD model). Each panel shows the coefficient of determination (R^2^), regression line intercept, and regression line slope. Red lines indicate linear regression lines. The data on the black lines indicate complete correspondence between the estimations by the two models.

A. B. C. 　D.


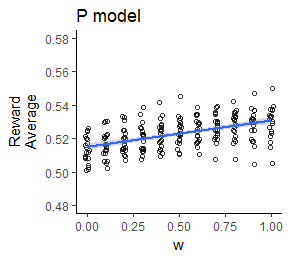

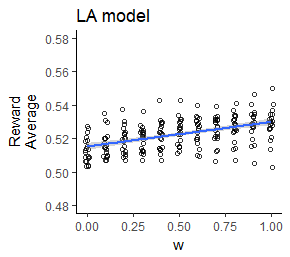

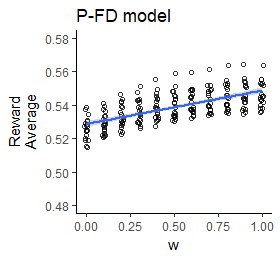

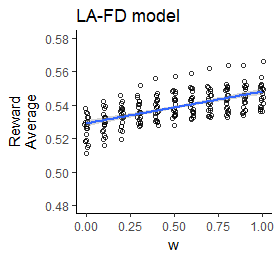


**Figure S3. Simulated results of the relationships between the weighting parameters and the average reward.** Using the models in which the parameter values were set at the medians of the estimated parameter values, reward averages for the task in this study were simulated. The true value of the weighting parameter $w$ was changed from 0 to 1 in steps of 0.05, and 20 simulations were conducted for each value. In the simulations, the reward in the task, which ranged from 1 to 9, was linearly transformed to a range from 0 to 1. The panels show the results of the simulations in **(A)** the parallel model (P model), **(B)** the parsimonious learning-rate adjustment model (LA model), **(C)** the P model with forgetting in which $\mu$ is a free parameter (P-FD model), and **(D)** the LA model with forgetting in which $\mu$ is a free parameter (LA-FD model). Gray areas indicate the 95% confidence intervals. We found positive correlations between $w$ and the average reward in every model (P model: *r* ­­= 0.52; LA model: *r* ­­= 0.49; P-FD model: *r* ­­= 0.66; LA-FD model: *r* ­­= 0.63). Note that these analyses were conducted to confirm the relationships between $w$ and the average rewards in the task setting of this study and not to compare the models because the sets of parameter values used in each model were different.

## Supplementary Tables

**Table S1. The reward settings in the current experiment.**

|  | All | C better | D better | Equal |
| --- | --- | --- | --- | --- |
|  | (n=253) | (n=111) | (n=110) | (n=32) |
| State C | 4.68 (2.36) | 6.28 (1.87) | 3.18 (1.74) | 4.28 (2.22) |
| State D | 4.72 (2.35) | 3.17 (1.74) | 6.42 (1.72) | 4.28 (2.22) |
| Δ (C-D) | -0.04 (3.37) | 3.11 (1.73) | -3.24 (1.67) | 0 (0) |

Note: This table shows the characteristics of the reward settings for the two-step task used in the experiment. The average rewards and standard deviations in states C and D and their average difference (Δ) are shown for all trials, including the trials where state C is better, the trials where state D is better, and the trials where states C and D produce the same reward. n represents the number of trials.

**Table S2.** **Information concerning the parallel models in which** $\boldsymbol{\alpha}_{\boldsymbol{L}}$ **and** $\boldsymbol{\lambda}$ **were set to one and the parsimonious learning-rate adjustment models in which** $\boldsymbol{\alpha}_{\boldsymbol{L}}$ **was set to one.**

| **Model** | **Basic model** | **Forgetting** | **Default value** | **Free Parameters** | **#** | **–LL** | **AIC** |
| --- | --- | --- | --- | --- | --- | --- | --- |
| P |  | - | - | $\beta, \pi, \rho, w$ | 4 | 3442 | 7117 |
| P-F0 | Parallel | o | Fixed (μ=0) | $\beta, \pi, \rho, w, \alpha_{F}$ | 5 | 3288 | 6867 |
| P-F05 |  | o | Fixed (μ=0.5) | $\beta, \pi, \rho, w, \alpha_{F}$ | 5 | 3066 | 6422 |
| P-FD |  | o | o | $\beta, \pi, \rho, w, \alpha_{F}, \mu$ | 6 | 3046 | 6441 |
| LA |  | - | - | $\beta, \pi, \rho, w$ | 4 | 3449 | 7130 |
| LA-F0 | Learning-rate adjustment | o | Fixed (μ=0) | $\beta, \pi, \rho, w, \alpha_{F}$ | 5 | 3295 | 6881 |
| LA-F05 |  | o | Fixed (μ=0.5) | $\beta, \pi, \rho, w, \alpha_{F}$ | 5 | 3065 | 6420 |
| LA-FD |  | o | o | $\beta, \pi, \rho, w, \alpha_{F}, \mu$ | 6 | 3046 | 6439 |

Note: This list provides information on the free parameters, the negative log likelihood (–LL) and the Akaike information criterion (AIC) summed over all participants (n = 29) for each model. The models differ regarding the basic model with parallel (P) or parsimonious learning-rate adjustment (LA). The models also differ in their forgetting-process assumptions: no forgetting, forgetting with a fixed default value (F05), or forgetting with a free default value parameter (FD).

**Table S3. Associations of estimated parameter values using the reduced models with psychopathology and other traits.**

|  |  | $\boldsymbol{\alpha}_{\boldsymbol{L}}$ | $\boldsymbol{\beta}$ | $\boldsymbol{w}$ | $\boldsymbol{\pi}$ | $\boldsymbol{\rho}$ | $\boldsymbol{\lambda}$ | $\boldsymbol{\alpha}_{\boldsymbol{F}}$ | $\boldsymbol{\mu}$ |
| --- | --- | --- | --- | --- | --- | --- | --- | --- | --- |
| *Correlation with OCI* | |  |  |  |  |  |  |  |  |
|  | P |  | **-0.45** | *-0.35* | **0.37** | -0.14 |  |  |  |
|  | LA |  | **-0.43** | **-0.39** | **0.37** | -0.12 |  |  |  |
|  | P-FD |  | **-0.39** | -0.14 | -0.14 | -0.15 |  | -0.23 | -0.12 |
|  | LA-FD |  | **-0.39** | -0.11 | -0.19 | -0.14 |  | -0.23 | -0.13 |
| *Correlation with STAI* | |  |  |  |  |  |  |  |  |
|  | P |  | 0.00 | 0.06 | -0.09 | 0.02 |  |  |  |
|  | LA |  | 0.01 | 0.08 | -0.10 | 0.01 |  |  |  |
|  | P-FD |  | 0.16 | 0.01 | 0.05 | -0.03 |  | *0.32* | 0.12 |
|  | LA-FD |  | 0.15 | 0.03 | 0.01 | -0.03 |  | 0.27 | 0.15 |
| *Correlation with SDS* | |  |  |  |  |  |  |  |  |
|  | P |  | -0.02 | 0.16 | -0.04 | -0.10 |  |  |  |
|  | LA |  | -0.01 | 0.15 | -0.05 | -0.11 |  |  |  |
|  | P-FD |  | 0.04 | 0.10 | 0.05 | -0.10 |  | **0.42** | 0.19 |
|  | LA-FD |  | 0.04 | 0.15 | 0.03 | -0.10 |  | **0.40** | 0.23 |
| *Correlation with PSS.10* | | |  |  |  |  |  |  |  |
|  | P |  | 0.03 | 0.24 | -0.26 | -0.14 |  |  |  |
|  | LA |  | 0.06 | 0.17 | -0.27 | -0.13 |  |  |  |
|  | P-FD |  | 0.11 | 0.23 | -0.29 | -0.17 |  | **0.42** | -0.03 |
|  | LA-FD |  | 0.11 | 0.29 | -0.31 | -0.15 |  | **0.41** | 0.00 |
| *Correlation with BIS11* | | |  |  |  |  |  |  |  |
|  | P |  | -0.21 | 0.29 | 0.10 | -0.18 |  |  |  |
|  | LA |  | -0.19 | 0.24 | 0.10 | -0.18 |  |  |  |
|  | P-FD |  | -0.09 | **0.54** | 0.00 | -0.25 |  | -0.09 | -0.04 |
|  | LA-FD |  | -0.09 | **0.57** | -0.01 | -0.23 |  | -0.11 | -0.02 |

Note: The questionnaires used in this study were the Obsessive-Compulsive Inventory (OCI) for obsessive-compulsive disorder, the trait portion of the State-Trait Anxiety Inventory (STAI) for trait anxiety, the Self-Rating Depression Scale (SDS) for depression, the Perceived Stress Scale (PSS) for stress, and the Barratt Impulsivity Scale 11th version (BIS-11) for impulsivity. The correlations of their scores with the model parameters are shown. Model parameters were estimated using the reduced models of the parallel model (P), the parsimonious learning-rate adjustment model (LA), the P model in which $\mu$ is a free parameter (P-FD), or the LA model in which $\mu$ is a free parameter (LA-FD). In the P and P-FD models, $\alpha_{L}$ and $\lambda$ were fixed to 1. In the LA and LA-FD models, $\alpha_{L}$ was fixed to 1. Italic: *p* < .10. Bold: *p* < .05.
